# Extreme precision in rhythmic interaction is enabled by role-optimized sensorimotor coupling: analysis and modelling of West African drum ensemble music

**DOI:** 10.1098/rstb.2020.0331

**Published:** 2021-10-11

**Authors:** Nori Jacoby, Rainer Polak, Justin London

**Affiliations:** ^1^ Research Group Computational Auditory Perception, Max Planck Institute for Empirical Aesthetics, Grueneburgweg 14, 60322 Frankfurt, Germany; ^2^ Music Department, Max Planck Institute for Empirical Aesthetics, Grueneburgweg 14, 60322 Frankfurt, Germany; ^3^ The Center for Science and Society, Columbia University, New York, NY 10027, USA; ^4^ Music Department, Carleton College, 1 North College Street, Northfield, MN 55057, USA

**Keywords:** sensorimotor synchronization, rhythm and timing, music performance, joint action, rhythm modelling, linear causal modelling

## Abstract

Human social interactions often involve carefully synchronized behaviours. Musical performance in particular features precise timing and depends on the differentiation and coordination of musical/social roles. Here, we study the influence of musical/social roles, individual musicians and different ensembles on rhythmic synchronization in Malian drum ensemble music, which features synchronization accuracy near the limits of human performance. We analysed 72 recordings of the same piece performed by four trios, in which two drummers in each trio systematically switched roles (lead versus accompaniment). Musical role, rather than individual or group differences, is the main factor influencing synchronization accuracy. Using linear causal modelling, we found a consistent pattern of bi-directional couplings between players, in which the direction and strength of rhythmic adaptation is asymmetrically distributed across musical roles. This differs from notions of musical leadership, which assume that ensemble synchronization relies predominantly on a single dominant personality and/or musical role. We then ran simulations that varied the direction and strength of sensorimotor coupling and found that the coupling pattern used by the Malian musicians affords nearly optimal synchronization. More broadly, our study showcases the importance of ecologically valid and culturally diverse studies of human behaviour.

This article is part of the theme issue ‘Synchrony and rhythm interaction: from the brain to behavioural ecology’.

## 1. Introduction

Complex human social behaviours often rely upon different members of a group taking particular roles or tasks. These roles can be assigned for a number of reasons: an individual's social status, charisma, expertise, innate ability and/or extra-personal resources may determine their role and position within a group; these reasons are not mutually exclusive. Many human social behaviours involve joint action(s) with varying degrees of temporal precision, from the sequence of chores in a kitchen to the precise timing of a sequence of passes on the soccer field [1,2].

Musical ensembles combine all of these aspects: social and musical determinations of seniority and status (leader/conductor versus section player; teacher versus student; soloist versus accompanist), specialized skills and expertise (drummer versus pianist versus violinist) and different roles as defined by the music (melody versus accompaniment). Moreover, the coordination among members of a musical ensemble is often highly complex in terms of its sequential and hierarchical temporal structure, and yet, it is also both highly precise and temporally flexible at the same time [3–5]. It is a form of interpersonal rhythmic ‘entrainment’ or sensorimotor synchronization, in which ensemble members employ adaptive timing mechanisms for error correction to compensate for both intentional and unintentional deviations from expected timing behaviours [3–13].

Our study examines a case of extreme temporal precision in musical performance, that of *jembe* drum ensembles from Mali. Malian drummers play complex, improvised rhythmic patterns at very fast tempos (up to 600 events per minute), near the rate limit for human performance while maintaining near-perfect synchrony [4,11,14]. In a recent study of synchronization in musical ensembles based on the cross-culturally broadest set of corpora available today [3,15], Malian drummers were found to have the lowest level of timing variability among groups studied (table 1), far lower than has been found in Western classical music performance, and slightly lower than other highly proficient Uruguayan candombe drummers [17]. How can Malian drummers maintain this high level of temporal coordination while playing complex improvised rhythms at such rapid tempos?

**Table 1.** Cross-cultural comparison of synchronization tightness. Pairwise ensemble asynchronies as reported in studies of musical genres from different geographical locations and cultures around the world. Following Rasch [16], pairwise asynchrony is the average of the root mean square (RMS) of pairwise differences between instruments articulating the same metric position.

data source	musical style	pairwise asynchrony (ms)
this study	*Jembe* drumming (Mali): 72 recordings of a single piece, ‘Suku’	17.0
Clayton *et al*. [3,15]	*Jembe* drumming (Mali): 16 recordings of three pieces, provided by author Polak	15.6
	*Candombe* drumming (Uruguay)	18.0
	*Son* and *Salsa* popular music (Cuba)	24.4
	*Stambeli* ritual music (Tunisia)	28.0
	*Raga* music (North India)	29.1
	*String quartet* (UK)	35.2
Rasch [16]	European chamber music	30–50

**Figure 1.**
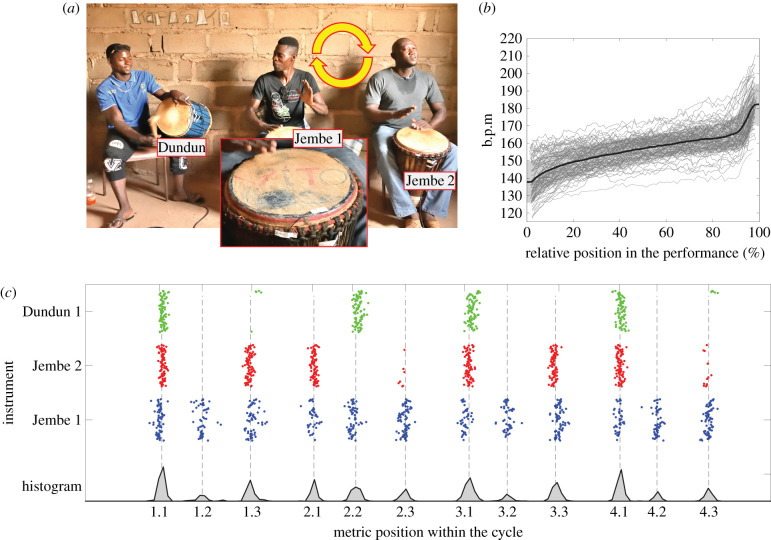
Jembe drum ensemble performance of Suku. (*a*) Main image: Jembe drum ensemble trio, comprising three players of instruments: Dundun (left): Modibo Diarra; Jembe 1 (centre): Antoine Ki; and Jembe 2 (right): Salifou Sulama. The two jembe players constantly switch instruments and roles (Jembe 1 and Jembe 2) after each take. Inset: Magnified detail of piezoelectric pickup taped to the membrane. (*b*) Plots of the tempo change over the course of each performance of Suku in the corpus. The thick black line indicates the grand average of all 72 performances; the grey-shaded area marks the average ± 1 s.d. (*c*) Upper tier: Distribution of all onsets within one performance of Suku, separated by instrument. *X*-axis, position within the rhythmic cycle; *y*-axis, position across all cycles in the performance. Lower tier: histogram of all onsets (aggregated across instruments) within one performance of Suku relative to the rhythmic cycle. Note that the metric positions within the cycle are non-isochronous.

A Malian jembe trio consists of three distinct musical roles, each assigned to a particular instrument and player: a virtuosic and highly variative lead role (Jembe 1), a short and simple unvarying accompaniment that projects the basic beat (Jembe 2) and a moderately variative ‘timeline’ pattern that is characteristic of a given piece (Dundun; figure 1; details in electronic supplementary material, §3.1). Here, we present a novel experimental paradigm for studying musico-social coordination, a controlled field experiment that exploits the conventional practice of role-switching among members of a drum ensemble: during their hours-long performances in the context of rituals and social celebrations, the lead and accompaniment jembe players will often switch roles. We collected data from 72 studio-recordings of a single piece, ‘Suku’ (see electronic supplementary material, figure S1) by four different jembe trio ensembles (16–22 trials or ‘takes’ per ensemble; see electronic supplementary material, table S1), with Jembe 1 and Jembe 2 players systematically switching roles; the Dundun player remained constant (figure 1*a*). Performances were recorded with piezoelectric transducers attached to each drum head (inset of figure 1*a*), yielding over 154 000 data-points for timing analysis (see §§4a,b).

Our corpus of audio recordings of musical performance combines four crucial aspects to an unprecedented degree: it is ecologically valid, capturing complex, high-quality ensemble performances of full pieces of real music; it is sufficiently large to allow for the application of data-hungry computational analysis; it has a stable metric pattern that allows us to perform automatic annotation and transcription from raw timing data (see §4c); and it allows us to study the timing relationships among ensemble members in terms of (i) the different musical roles of each instrument, (ii) differences among individual players, and (iii) differences among different ensembles.

The aims of the study are four-fold. First, to document the relative contribution of musical role, individual and group differences to tight synchronization despite large overall tempo changes. Second, to precisely document the degree to which each member of the ensemble responds to timing deviations made by other members of the ensemble, that is, the pattern of ‘error correction couplings’. Third, to show whether and how this coupling pattern is related to both the specific musical role of each drum in the ensemble and any idiosyncratic differences among particular performers or groups of performers. Fourth and last, by modelling alternative error-correction coupling patterns in simulated performances of Suku, to determine what the optimal role-specific coupling patterns for jembe trios would be and then compare them with the coupling patterns we have observed in our data. Our broader aim is to document how temporal precision in human joint action depends on both the differentiation and optimal coordination of social–musical roles.

Many musical factors, such as pulse clarity, event density, onset perceptibility, musicians' skills, musical practices (e.g. rehearsal prior to performance, improvisation during performance) and style-specific aesthetic ideals, all can contribute to the degree of precision in ensemble synchronization. For example, the crisp onsets produced by drum-strokes in contrast with other types of instrument sounds may constitute a precondition to a particularly high degree of precision, which may be reflected in the fact that the most precise synchrony hitherto documented involves either percussion ensembles (Malian jembe and Uruguayan candombe) or percussion-heavy music (Cuban son; table 1). Relatedly, percussionists often outperform non-percussionist musicians in rhythm perception skills, particularly in the context of sensorimotor synchronization tasks [18]; the state of research on this issue is ambivalent, however [19]. Another relevant factor is the performers’ desire for tight ensemble synchrony: while it is an aesthetic ideal characteristic of certain African and African-diasporic musical genres, including jembe and candombe music [20], it is not necessarily universal. Other musical genres may intentionally prefer a certain degree of looseness or fluctuation in ensemble synchronization (for example, Noh theatre music from Japan [21]).

We therefore took the approach of a case study of one specific style of music, and in this context decided to measure naturalistic ensemble music performances that fully articulate these factors, in contrast with more constrained performances or simplified tapping experiments where the material is highly reduced in complexity [11]. Our experimental design focuses on how such precision is possible in relation to the individual and socially interactive behaviours that constitute the Malian jembe ensemble's performance (figure 1). In particular, we study differences between individual players (e.g. in expertise and/or seniority), between ensembles (teams of individual players) and between musical roles (e.g. lead versus accompaniment), which differ with respect to their inherent complexity and variability.

## 2. Results

### (a) Factors influencing synchrony in ensemble performances

Suku, like most jembe drum ensemble pieces, is characterized by a steady tempo increase over the course of a performance (figure 1*b*; see electronic supplementary material, figure S2 for tempo curves for each ensemble configuration). Likewise, the musical roles of the Dundun and Jembe 2 involve highly repetitive rhythmic patterns that allow for the automatic identification of the music's cyclic temporal grid (electronic supplementary material, figure S3). Figure 1*c* shows that relative onset locations are clustered into 12 specific positions within the cycle. This corresponds to a four-beat metre, with each beat comprised of three slightly uneven subdivisions [22]. Eighty-five per cent of the drum-stroke onsets occur in windowed temporal positions that collectively constitute less than 25% of the cycle span, as is visible in the histogram of the distribution onset locations within the cycle (figure 1*c*, lower tier). Thus, any given event can be indexed relative to its position within the ‘local’ metric cycle (electronic supplementary material, figure S3; details in §4c in the Methods section).

**Figure 2.**
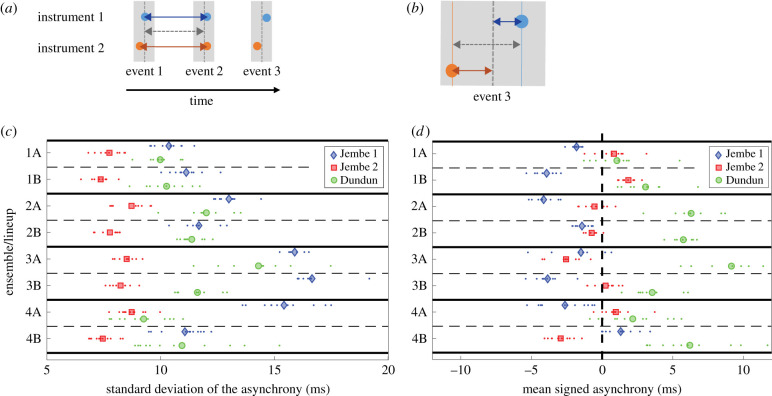
Onset asynchronies relative to the metrical grid. (*a*) Schematics of events and inter-onset intervals (IOIs). Event onsets played by different instruments are marked with differently coloured circles. IOIs between adjacent events within the same instrument are marked with coloured arrows. Vertical grey-shaded bars outline the metric grid positions; dashed dark grey lines within the bars mark the mean location of onsets within each position. (*b*) Schematics of asynchronies between adjacent event onsets and between event onsets and respective metric grid location. (*c*) Standard deviation of onset asynchronies in Suku. The asynchronies were computed for each instrument separately with respect to grid locations, organized by ensemble/lineup; each data point = average s.d. within a performance. (*d*) Mean signed asynchronies in Suku, organized by ensemble/lineup; each data point = average signed asynchronies within a performance.

Our experimental design is specifically equipped to test the relative contribution of three factors in maintaining ensemble synchrony: ‘Ensemble’ indicates the four different jembe trios; ‘Lineup’ differentiates the trials where the Jembe 1 and Jembe 2 players switch roles in half of the trials, and ‘Role’ indicates the musical role of each instrument (Jembe 1, Jembe 2 and Dundun); for details, see §4a. The dependent variables were the standard deviations and signed means of asynchronies, the latter defined as the difference between produced onsets and their expected grid locations (figure 2*a,b*; electronic supplementary material, figure S3*b*). Figure 2*c,d* shows these standard deviations and mean signed asynchronies across all ensembles and lineups per ensemble. Each Role displays a characteristic asynchrony (Jembe 1 is early, Jembe 2 is onbeat, Dundun is late) and variability (Jembe 2 is less variable than Jembe 1 or Dundun) across conditions (Ensemble and Lineup). However, the differences between instrumental roles are nonetheless extremely small in magnitude—on the order of 5–10 ms. Indeed, they are only detectable because the overall timing stability and precision within each individual part provides a reference framework against which even very small timing differences can be salient.

We used mixed-effect modelling implemented with the *fitlme* function in Matlab [23] to measure the relative contributions of Ensemble, Lineup and Role in maintaining ensemble synchrony. As performance criteria, we used Bayesian information criterion (BIC), Akaike information criterion (AIC), log-likelihood [24] and explained variance of the model predictions. Following an exploratory analysis, we created a model with Lineup as a fixed effect and Ensemble and Role as random effects, using the standard deviation of the asynchrony as our dependent measure (see electronic supplementary material, §3.2). This model explained 72.0% of the variability of the data. We compared the initial model to the best performing model obtained by performing a search over a reduced number of factors. We found that omitting the Lineup or Ensemble factors resulted in a small and modest change to the performance criteria, respectively (omitting Lineup: ΔBIC = 11.5, ΔAIC = 70.1, Δlog-likelihood = 5.7, nearly no change in explained variance; omitting Ensemble: ΔBIC = 70.1, ΔAIC = 74.0, Δlog-likelihood = 38.0, explained variance = 57.3%). By contrast, we found a large change when omitting the Role factor (ΔBIC = 212.3, ΔAIC = 215.7, Δlog-likelihood = 108.9, explained variance = 16.3%). We repeated the same analysis with the mean onset asynchrony as the dependent variable (figure 2*d*) and obtained similar results. Specifically, the model explained 57.4% of the variance, and omitting the Lineup or the Ensemble factor results in small change approximating the optimal model (ΔBIC = 12.8, ΔAIC = 9.5, Δlog-likelihood = 3.7, nearly no change in explained variance), whereas omitting the Role factor results in a large degradation of performance measures (ΔBIC = 155.9, ΔAIC = 162.7, Δlog-likelihood = 83.3, explained variance = 0.4%). Taken together, these results suggest that mean and standard deviation of the asynchrony are largely determined by Role, and not by Ensemble and/or Lineup.

To validate the BIC and AIC scores, we also performed a complementary analysis that does not rely on a mixed-effect approach. In this simple analysis, we compared the percentage of explained variance obtained by the dependent variable (regressing the mean or standard deviation of the asynchrony) with a single categorical factor, i.e. we replaced each data point with group averages determined by the factor category. Here, again, we found that Lineup and Ensemble explained far less variance compared with Role: for standard deviation of asynchrony, Lineup, Ensemble and Role covered 2.1%, 15.6% and 56% of the variance, respectively; likewise for mean asynchrony, Lineup, Ensemble and Role covered 0.6%, 2% and 57.2% of the variance, respectively.

Finally, we then repeated the same analysis with two additional dependent measures, namely mean and standard deviation of the inter-onset intervals (IOIs). The result showed similar trends (see electronic supplementary material, §3.2). In sum, the role of each musical instrument (Role) is much more important than the characteristics of individuals (Lineup) or groups of musicians (Ensemble).

### (b) Understanding coupling structure with linear modelling

To maintain synchrony, a musician must continuously attend to small deviations in the onset timings produced by other musicians and adapt to them [11,25–28]. These deviations need not be consciously detectable in order to elicit a phase correction response [29], and when these responses are present one can speak of ‘couplings’ among the musicians in an ensemble. Previous literature [13,30,31] examined music where the rhythm is largely homogeneous across all instruments in the ensemble. However, the rhythmic texture of Suku is more complex, as it comprises distinctly different rhythmic patterns distributed across ensemble members; every musician does not articulate every position in the rhythmic cycle. Therefore, we compared actual IOIs to the expectations based on their prototypical durations, based upon their average onset positions within the rhythmic cycle (figure 2*a,b*), from which the metrical grid can be empirically inferred. Here, we relied on the fact that Malian drummers are extremely stable with respect to their relative phases (figure 1*c*) within the cycle despite the large tempo changes characteristic of this repertoire (figure 1*b*). Formally, this model can be written as

**Figure 3.**
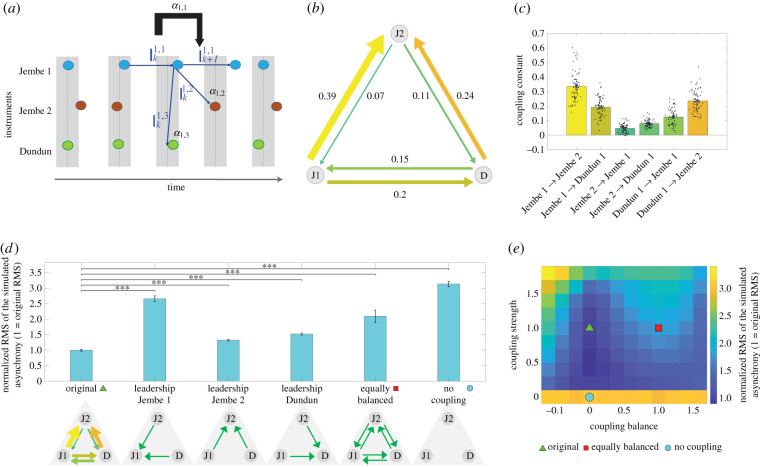
Measured and simulated coupling between ensemble members. (*a*) Schematic of coupling among individual instruments in the ensemble (*α* coefficients). Coloured circles indicate event onsets. Vertical grey-shaded bars outline the metric grid positions; dark grey lines mark the mean location of onsets within each position. *I^i,j^_k_* indicates the IOI between instruments *i* and *j* at position *k; α^i,j^* depicts the coupling constant associated with instrument *i* and *j*. (*b*) Measured couplings averaged across all performances of Suku: direction of arrows indicates the influence and influencer instruments, namely the tendency to follow (adapt to) the indicated instrument; thickness and colour of the arrow indicate coupling strength. (*c*) Measured coupling constants for pairs of instruments. The bars indicate the mean value. The colouring of the bars corresponds to the colour of the respective pairs of instruments (arrows) in (*b*), and dots indicate individual trials, randomly displaced horizontally for visual clarity. (*d*) Simulation results. A comparison of coupling models; *y*-axis indicates the root mean square (RMS) of simulation asynchrony averaged across all instruments. Values linearly scaled so that the original condition will have a normalized value of 1. Error bars represent 1 s.d. obtained by bootstrapping. Asterisks indicate statistical significance (***, *p* < 0.001). (*e*) Simulation results. The actual coupling pattern is marked with the green triangle. Simulated couplings indicated by the red square (original strength and equally balanced coupling) and by the blue circle (zero strength and original/actual balance). Colours in the heatmap represent the RMS of the asynchrony. Values linearly scaled so that the original condition has a normalized RMS of 1.

2.1 $$I^{i,i}{\;}_{k+1\;}=\alpha_{i,i}I^{i,i}{\;}_{k}+\Sigma_{j\neq i}\alpha_{i,j}I^{i,j}{\;}_{k}$$

where *I^i,i^_k+_*_1_ is the adjusted IOI at onset *k* *+* 1, and $I^{i,j}{\;}_{k\;}$ is the inter-onset difference between onset *k* in instrument *i* and the onset that precedes onset *k* *+* 1 in instrument *j*, *α_i,i_* is the influence of the previous inter-onset difference of the same instrument *i* and *α_i,j_* is the influence of instrument *j* on the inter-onset difference of instrument *i* (figure 3*a*). Note that to account for the complex texture, we used here adjusted IOIs, namely how ‘elongated/late’ or ‘shortened/early’ a given duration is compared to the prototypical durations relative to the entire cycle duration ($I^{i,j}{\;}_{k\;}=\;J^{i,j}{\;}_{k\;}-{\bar{J}}^{i,j}{\;}_{k\;}$*,* where $J^{i,j}{\;}_{k\;}$ and ${\bar{J}}^{i,j}{\;}_{k\;}$ are the raw and prototypical average durations, respectively; see electronic supplementary material, §3.3 for details). Despite its relative simplicity, the model captures 66% of the explained variance in IOIs.

Note that coupling is dependent on two instruments: the ‘influencer’ and the ‘influenced’ (figure 3*b*). We therefore incorporated these two factors (Influencer instrument, Influenced instrument) into a mixed effect model as random effects, with Ensemble also as a random effect and Lineup as a fixed effect, as per our analysis of the mean and standard deviation of the asynchrony given above (see electronic supplementary material, figure S4 for average coupling constant values, separated by ensemble and lineup). This model explained 58% of the explained variance. We validated the choice of this model by comparing it to the performance of alternative models and we found that it was nearly optimal (ΔBIC = ΔAIC = 0.71, Δlog-likelihood = 0.36, nearly identical explained variance compared with the optimal model; see electronic supplementary material, §3.2). We then considered all other possible models with a reduced number of factors. Omitting the Lineup and Ensemble factors resulted in a small change to the performance criteria, respectively (omitting Lineup: ΔBIC = ΔAIC = 0.72, Δlog-likelihood = 0.36, explained variance = 57.3; omitting Ensemble: ΔBIC = 2.4, ΔAIC = 6.4, Δlog-likelihood = 4.2, explained variance = 55.6). By contrast, we found a large change when omitting the instrument factors (omitting Influencer instrument: ΔBIC = 102.2, ΔAIC = 110.4, Δlog-likelihood = 57.2, explained variance = 42.1; omitting Influenced instrument: ΔBIC = 133.7, ΔAIC = 141.8, Δlog-likelihood = 72.9, explained variance = 37.7). We also performed a complementary analysis that does not rely on a mixed-effect model and found that, similarly, Ensemble, Lineup, Influencer instrument and Influenced instrument constituted 1.8%, 0.11%, 37.5% and 41.9% of the explained variance, respectively; Influencer and Influenced together explained 57.3% of the variance. Together, these results suggest that Influencer instrument and Influenced instrument are the main contributors to coupling strength, far more than Ensemble and Lineup.

### (c) Optimal coupling patterns

Two obvious strategies for ensemble coupling are either (i) a single musician serves as the ‘leader’ and others adapt their timing to that musician, or (ii) error correction is distributed equally across all members of the ensemble. Evidence of both the former [5,32–36] and the latter [13,25,30,31,37] has been found, though these studies also show that neither strategy is operative in its most basic form. Figure 3*b,c* shows the average coupling pattern over our entire corpus of 72 recordings of Suku. We found that it is not the lead drum (Jembe 1) but the non-variative accompaniment drum (Jembe 2) that exerts the strongest influence on the other instruments in the ensemble (*p* < 0.001 via paired *t*-test, Bonferroni correction applied). At the same time, Jembe 2 is itself influenced by the other instruments only to a small extent; the Jembe 1 and Dundun mutually influence each other to an approximately equal extent. Thus, the pattern of coupling distribution can be characterized as asymmetric, yet at the same time relatively balanced rather than strictly hierarchical. Figure 3*b* depicts the degree and direction of mutual influence for each instrument, where the thickness and colour of the arrows show the coupling strength. Figure 3*c* shows the individual coupling constant for each trial as well as the means for each instrument pair. Note that the coupling constants are on average positive.

Our results are in accord with qualitative research in jazz performance, which suggests that ensemble synchronization can be anchored by the accompaniment parts of the ensemble (i.e. the ‘rhythm section’) rather than leading/soloing instruments [38,39]. Our Malian musician participants' own understanding of the different roles in the jembe ensemble are also in accord with this perspective. Author Polak performed a series of interviews with the musicians and found that they acknowledged that the lead drum (Jembe 1) is the most highly variative, and is hence a less reliable guide for ensemble coordination. In the words of Sedu Keita, jembe player in Ensemble 1: ‘The dundun and the second jembe player need to go along in close contact with each other; because if the dundun or second jembe accompanist tries to listen to the lead drummer, you will play crap; you will get lost’. The musicians also acknowledged that the less variative and sparser second jembe and dundun are specifically tasked with ‘keeping the time.’ From dundun expert Draman Keita's (Ensemble 2) perspective: ‘The second jembe and the dundun go along together … The soloist again will take on the time from both the second jembe and the dundun. The dundun player will listen to the second jembe accompanist so that he is able to keep the regular track. I [the dundun player] will keep track of my time, because when I lose my time, the others, too, will go astray’ (see electronic supplementary material, §4 for extended quotations in both the original source language and English translation).

Testing the musicians' self-reports, we modelled a series of simulated musical performances in which the coupling patterns were systematically manipulated, yet where the density and variability of each instrument were derived from actual performance data. We compared the following simulations: (i) the original coupling pattern as measured in the recorded performances, (ii) three variants of hierarchical leadership, each with a single instrument as the leader and the two other instruments as followers, with no other couplings among them, (iii) equally distributed coupling (balanced between and across pairs of instruments, i.e. ‘democracy’), and (iv) no coupling at all, involving complete independence of instruments (see electronic supplementary material, §3.4 for more details). We found the original coupling pattern shows the lowest level of asynchrony/error among the modelled variants (figure 3*d*). While setting the lead instrument (Jembe 1) to be the leader results in very large synchronization error, assigning leadership to either the Jembe 2 or the Dundun also produces synchronization errors significantly larger than the original pattern, albeit substantially smaller than when Jembe 1 is the leader. Importantly, an equally balanced distribution also generates a substantially larger synchronization error than the original (*p <* 0.001 via *t*-tests; Bonferroni correction applied). Not surprisingly, no coupling at all (complete independence, where all onsets are determined by prototypical metric locations with independent random noise) results in the highest levels of asynchrony.

To further study the effects of different error correction strategies beyond the selected hypotheses, we explored a two-dimensional parameter space wherein we continuously manipulated the total amount of coupling strength (*y*-axis) and its allocation among the members of the ensemble from balanced to unbalanced (*x-*axis). In figure 3*e*, we arbitrarily positioned the no-coupling model (with zero coupling matrix) at the origin (0,0) marked as a circle, the original/actual coupling model at (0,1) marked as triangle, and the democratic model (with all coupling constant equal) at (1,1) marked as a square (see §4e and electronic supplementary material, §3.4 for additional details). We found that the location of actual data simulation error was not significantly different from the optimal location (i.e. where root mean square (RMS) of the simulated asynchrony is lowest) within the parameter space (*p =* 0.47 via Wilcoxon rank-sum test). Specifically, the optimal location involves the same coupling structure but with slightly reduced coupling strength. This is consistent with the idea that Malian drummers, through sustained performance practice over both their individual musical development and broader, collective stylistic evolution, have discovered the optimal attentional/coupling strategy for each part of the ensemble.

Finally, we performed a number of control analyses to assess the robustness of our findings (electronic supplementary material, §3.5). First, we compared our results with another established method of coupling analysis, namely, Granger causality, which measures causal influence within two time-series signals [33,34,40–42]. We found that this approach delivers similar results to our basic analysis method (electronic supplementary material, figure S5*a–c*). Then we tested whether the results would be different if instead of using the absolute and thus tempo-dependent durations of IOIs, we detrended the data and based our analysis on phase differences. Here, too, the results were very similar to the main approach (electronic supplementary material, figure S5*d–f*), suggesting that tempo changes scale-up, but do not qualitatively alter the consistent differences between the musicians' phases. Next, we tested whether the results are similar if we use asynchronies rather than IOIs as data for the coupling analysis (for comparison of these methods, see review in [43,44]). We found that while the coupling constants are numerically different, the overall pattern of results yielded from these earlier models was similar to ours. For instance, Jembe 1 strongly adapting to Jembe 2 is the strongest coupling relation in both analyses (electronic supplementary material, figure S5*g–i*). We then explored whether the results would be different when adding complexity to the linear model by considering a longer past history, namely a higher-order linear model [43]. Again, the results were similar to the main analysis (electronic supplementary material, figure S5*j–l*). Finally, we explored whether the coupling changes substantially during the piece. Since reliable computation of coupling constants requires a considerable amount of data, and thus can only be performed on large segments of the data per recording [44], we tested whether the coupling constant changes for the first versus the second half of the piece. As in the other control analyses, we found the coupling constant to be very similar across both sections, suggesting that the synchronization behaviour does not change substantially in the course of performance (electronic supplementary material, figure S5*m,n*). In summary, the four different control analyses show that our approach to modelling the mutual adaptation relationships between the different instruments in the ensemble is robust.

## 3. Discussion

In this paper, we document the extreme rhythmic precision in Malian drum ensemble performance, characterized by their exceptionally low temporal variability and very high degree of synchrony. We collected performance data from four distinct ensembles in which the two jembe drummers systematically switched roles (lead versus accompaniment). These data allow us to assess the effect of individual players, ensembles and musical roles on ensemble synchronization (figure 1). Across three measures—means of the onset asynchronies, standard deviations of the onset asynchronies and coupling constants—we found that the dominant factor is musical role; individual players and differences among ensembles contribute much less to explaining the observed behaviour (figure 2). Patterns of small microtiming variation were then used to identify causal temporal coupling relations among the group members (figure 3*a–c*). Small differences in synchrony and variability can be related to differences in coupling associated with specific musical roles—highly variative lead drum (Jembe 1), invariant time keeper (Jembe 2) and near invariant timeline (Dundun)—and their mutual relationships. Their complex dynamic is consistent with the self-reports of the musicians as documented in post-experimental interviews.

Our simulations of hypothetical coupling arrangements suggest the Malian musicians have developed a near-optimal coupling strategy for their particular ensemble, an ensemble in which musical roles differ in terms of their information density and variability. In particular, an asymmetrically distributed model (derived from what we observed in the real performances) performs substantially better than both an equally balanced (democratic) and a hierarchical dominance model that ascribes full leadership to the so-called mother-drum or lead drum (Jembe 1); note that in terms of artistic interaction and communication (and often also socio-economic organization) the lead drummer actually does play the most dominant social role in the ensemble, comparable to the first violin in European chamber music (figure 3*e*). Our study thus provides clear demonstration that the core component of the human ability for temporal coordination of rhythmic behaviour, namely, error correction [11,45], can be optimized in context- and task-specific ways in complex, real-world joint action.

The roles in a musical ensemble are not simply musical roles, but social roles as well—making music together is a social activity [46–48], especially for and among the players involved. The key dimension of contrast/variation along which Malian ensemble coordination is organized is not leadership/followership (i.e. hierarchical dominance) but the extreme differentiation of the behavioural repertoire(s) that define the musical roles and the social interaction among them. Jembe 1 functions not only as the primary ‘melodic’ instrument and leading ‘voice’ in the ensemble; its improvisatory character is grounded in its need to flexibly control the participatory and interactive aspects of the performance (individual and group dancing, active audience response, ritual action) that are the core functions of the social occasion where these performances occur. By contrast, Jembe 2 is not allowed the variation of a single note of its pattern; the Dundun must constantly present the signature ‘timeline’ that identifies the piece as Suku and orients Jembe 2's pulses within the metric cycle. The coupling patterns we have uncovered, in addition to engendering the extreme rhythmic precision we have documented, are also reflections of the outward (Jembe 1) and inward (Jembe 2 and Dundun) social orientations of these different musical roles.

While our study attempted to collect data in a context that is both experimentally controlled and at the same time ecologically valid, our experimental method misses some important components of real-world jembe drum performance. Malian drumming occurs usually in the context of dancing and singing, both of which were absent during our data collection. Future research should apply techniques such as motion capture or machine learning video annotations to measure dance–music interactions. In addition, in our experimental trials, each ‘take’ ran from 2 to 3.5 min; in real-world contexts, performances are open-ended and can last from 2 to 20 min. In terms of our analysis of causal temporal relations, we focused on linear models with first-order statistics assuming uniform coupling over the course of the metric cycle. While our control analyses supported our approach (see electronic supplementary material, figure S5 and related discussion, electronic supplementary material, §3.5), indicating that higher-order models and other alternative analytical approaches do not substantially change the results, additional models, including alternative higher-order models such as differential coupling constants across metric positions within the cycle should be considered.

Our research illuminates a system of ensemble synchronization where simple accompaniment parts, rather than solo/leading parts (e.g. a jazz soloist, the first violin in a string quartet, an orchestra's conductor or the lead/master drummer in a West African percussion ensemble), consistently serve as the core timing reference to which other ensemble members adapt. This system of accompaniment-based timekeeping has been qualitatively described in jazz and other groove-based or dance-oriented musical genres. However, previous empirical research in ensemble synchronization has focused on ensembles that do not feature this kind of musical structure, specifically piano duos and string quartets from European art music traditions, and as a result has come to rather different conclusions regarding role-distribution in ensemble synchronization. Thus, while studies of ensemble synchronization in string quartet performance found evidence of asymmetric mutual couplings among players (rather than strictly hierarchical leadership), they also showed that string quartet performance at least partly involves adaptation to the presumptive leading role, that is, the first violin [13,30,31]. This stands in contrast with the consistently minimal adaptation to the lead drum and (conversely) consistently strong adaptation to the accompaniment roles we found in jembe performance. This speaks of two qualitatively different approaches to ensemble synchronization. Thus, our results underscore what can be gained from studying participants and cultural performances or artefacts beyond the laboratory in the Western World. Focusing solely on WEIRD groups (Western, Educated, Industrial, Rich and Democratic [49,50]) limits what one can observe and understand regarding human creativity and ability. A first step towards overcoming this cultural sampling bias is the integration of humanistic methods (in our case, ethnomusicological thinking and expertise) into scientific research. The ethnomusicologist member in our team of authors, Polak, proposed the research idea for the present study based on his practice-based knowledge of ensemble performance processes, and our data collection was only possible owing to the social network of musicians and research partners in Mali that he has cultivated over three decades of research. Polak was also able to obtain the musicians' qualitative assessments of our quantitative observations (see electronic supplementary material, §4), which paralleled our findings and modelling. Our work would be even better were we able to have our Malian colleagues involved in the conception of our research questions and hypotheses, and in the analysis of our data; we hope and aim for them to be more centrally involved in our future research. What our collaboration with Malian musicians has shown is that the study of behaviours at the limits of human abilities can give us a clearer perspective on the mechanisms that underlie those abilities. Understanding those behaviours, and the mechanisms that underlie them, can show the full range of possibilities for human perception and action coordinated in time and across individuals.

## 4. Methods

### (a) Corpus design and data collection

Author Polak produced the corpus of recordings in Bamako, the Malian capital, in February 2016. Recording, raw data and processed data are available in an OSF repository: https://doi.org/10.17605/OSF.IO/8WYAV. Four distinct trio ensembles, consisting of 12 urban professional drummers, were hired and paid for 1 day of studio work. Each of the four recording sessions (one session per ensemble) involved about 2 h of playing time. With technical set-up, atmospheric preparations including conversation and the consumption of food and tea, information about and consent to the research, the recording itself and post-experimental interviews, each session lasted for 5–7 h. All musician participants gave written informed consent, in accordance with the Declaration of Helsinki.

The drummers brought their own instruments. Piezoelectric transducers (K&K Sound Hot-Spot) were attached to the skin of each drum, very close to the rim where it does not compromise the vibration behaviour of the membrane and thus the sound (figure 1*a*). The relatively clean signal of each pick-up was recorded into a separate channel of a portable 4-track digital studio (Roland R44), which afforded simple and accurate automated onset detection.

In each ensemble, one musician specialized in playing the Dundun, while the two jembe players were proficient as both lead drummers and accompanists. In Mali, musicians tend to be aware of, and pay respect to, their relative seniority. This was relevant for the two jembe players in each trio ensemble, even though the degree of differentiation (range of variability) was small. For example, Sedu Keita, the player of Jembe 2 in Ensemble/Lineup 1A (born 1964) did his apprenticeship with Drisa Kone, the player of Jembe 1 (born 1960) four decades ago, but in the past 30 years both of them worked independently as master drummers. In each of the four recording sessions (one per ensemble), the more senior of the two jembe players in a trio would start out, by self-selection, with playing the lead drum role (Jembe 1), which is always (both live and in studio situations) placed in the centre of the ensemble. After the first take, the two jembe players changed seats, instruments and musical roles (figure 1*a*). Thereafter, the two jembe players continued with role-switching after each of the 16–22 takes that we recorded per ensemble. This systematic alternation of roles was motivated by our interest in the relative contribution of individuals when compared with ensembles and instrumental roles, and was performed by the players upon our request. We refer to the swapping of Jembe 1 and Jembe 2 as the variable ‘Lineup’. In each ensemble in the corpus of recordings, Lineup A denotes that the senior jembe player is performing the lead drum (Jembe 1), whereas Lineup B indicates that the senior jembe player is providing accompaniment (Jembe 2).

### (b) Onset detection and markup

Each of the 72 takes in our corpus lasted between 104 and 282 s (average 162 s); the total running time of the corpus is 201 min (see electronic supplementary material, table S1). We used the software ‘Sonic Visualizer’ with the plugin ‘Onsets DS’ to automatically detect note onsets, accurate to ±2 ms [51,52]. False alarms (one sound event being registered as two or three onsets) were filtered by discarding onsets following an initial onset within a 50 ms window. This threshold of 50 ms was chosen based on the fact that the minimal inter-onset interval between metric events in our corpus, at the fastest tempos included, is about 100 ms. The filtered time-series were reimported to an audio-editor and checked by visual inspection.

### (c) Data preparation: metric annotation of onsets

The jembe performances in our corpus exhibit the large-scale tempo accelerations characteristic of this repertoire, starting at initial rates of approximately 120–160 b.p.m. to final speeds of approximately 160–200 b.p.m. over the course of the piece (see electronic supplementary material, figure S2). However, as shown in figure 1*c* and electronic supplementary material, figure S3, the phase positions of each drum-stroke (i.e. their relative locations within the metric cycle) are extremely stable across tempo changes. We established a process that uses this property of the music to automatically annotate the metric positions for all onsets of the entire piece. First, we identified the ostinato pattern played by Jembe 2, which articulates each beat including the turning point (downbeat) of every cycle. We then identified the onsets articulating each downbeat in all other instruments as onsets within a window of ±4.5% of the cycle duration defined by Jembe 2 alone. This threshold was chosen because it distinguishes the downbeat from adjacent metric positions, each of which covers 8.3% of the cycle duration. We then averaged the event onset locations of all instruments that articulated a given downbeat and re-computed the cycle durations on that basis. Within this revised framework, we calculated the phases associated with other onsets as the relative location within each cycle, now calculated based on downbeats of all ensemble members. Electronic supplementary material, figure S3A presents histograms of these phases and it is apparent that they are concentrated in 12 narrow clusters corresponding to each of the non-equal subdivisions within the 4-beat cycle [22,53–55]. We then computed the mean position (relative to the metric cycle duration) of each cluster, and used these prototypical locations as a virtual metric grid. We assigned each onset to the closest metric grid position according to its phase (electronic supplementary material, figure S3B). We discarded from further analysis all onsets located outside a symmetric window of 24% of the beat duration for each position. In the case of an ornament with two onsets within the same position, we considered only the onset closest to the prototypical location. The result of this process was an assignment of every remaining onset to a single metric position.

The total set of raw data-points consisted of 158 263 drum-stroke onsets. Trimming the beginnings and endings of pieces, where ensemble coordination is unsettled and not all instruments are present amounted to a loss of 3% of the onsets. The filtering of onsets involved a further loss of 1.8% of the raw data. The final corpus comprises 150 717 data-points.

### (d) Model fitting

To obtain the results of figure 3, we employed the following model. This equation was obtained by rewriting equation (2.1) with explicit residual noise and intercept terms that are fitted separately for each instrument:

4.1 $$I^{i,i}{\;}_{k+1}=\alpha_{i,i}I^{i,i}{\;}_{k}+\Sigma_{j\neq i}\alpha_{i,j}I^{i,j}{\;}_{k}+\alpha_{0\;}^{i}+n^{i}{\;}_{k}$$

where $\alpha^{i}{\;}_{0\;}$ is an instrument-specific intercept term, $n_{k}^{i}$ ∼ $N(0,\sigma_{i})$ is an unbiased Gaussian residual noise with variance $\sigma_{i}^{2}$, and coupling constant and IOIs are as in equation (2.1) (figure 3*a*; see derivation and further modelling detail in electronic supplementary material, §3.3). The parameter fitting was done separately for each performance trial (take). Electronic supplementary material, figure S4 shows the averages across performances by each ensemble and lineup. To help in the visualization, we normalized the colour and arrow width within each plot so that the largest coupling is always represented with the brighter colour and widest arrow; numeric values are also displayed. We then computed the averages across all performances, as shown in figure 3*c*, which displays the individual coupling constant for each pair of instruments for each performance in our corpus (dot = one performance, random jitter added to the *x*-axis to for visualization purpose) and averages (bars) along with the standard error of the mean (error bars). Note that we generally omitted from the graph the self-coupling constants and intercepts even though they were computed within the model. The self-coupling constants were all negative (averages were −0.18, −0.56 and −0.47 for Jembe 1, Jembe 2 and Dundun, respectively).

### (e) Simulations

We generated 500 simulations for each coupling model. We started with the real data, and computed the model fits for this particular performance. We then generated simulated data based on equation (4.1) where in each simulation, we kept the original residual noise magnitude intercept and coupling constant, forming the ‘original coupling’. In other words, in each simulation, the input was a set of coupling constants. We kept the music texture (which metric onsets are articulated) and the overall variability of the asynchrony associated with each instrument. We then simulated artificial data where the deviations from the metric grid were determined by the model of equation (4.1), replacing the coupling constants with the simulated values. The variability associated with each instrument determines the variability of the Gaussian noise term $n_{k}^{i}.\;$ After randomizing the noise term, we used equation (4.1) and the given coupling constants to compute the simulation IOIs for all articulated onsets. We measured the degree of overall variability averaged across instruments for each set of simulations with different structures of coupling matrices.

We created alternative coupling models (as explained in the electronic supplementary material, §3.4) by replacing the original coupling matrix $\alpha_{i,j}$ with five alternatives

$$\alpha^{J1}{\;}_{i,j},\;\;\alpha^{J2}{\;}_{i,j},\;\;\alpha^{D}{\;}_{i,j},\;\;\alpha^{\mathrm{balanced}}{\;}_{i,j},\;\;\mathrm{and}\;\alpha^{\mathrm{no}}{\;}_{i,j}.$$

These correspond to Jembe 1 (J1) as the leader, Jembe 2 (J2) as the leader, Dundun (D) as the leader, equally balanced (‘democratic’ coupling), and no coupling whatsoever. For each matrix, we computed the simulated RMS of the model compared with the metric grid. We then averaged the results across the 72 simulations. We plotted the RMS of the asynchrony, and to help the comparison of the different conditions, we linearly scaled all values so that the original condition will have a normalized value of 1 (figure 3*d*—note that the error bars represent 1 standard deviation obtained by bootstrapping).

To further study the effects of different phase correction strategies, we explored a two-dimensional parameter space wherein we continuously manipulated the phase coupling strength (*y*-axis) and its allocation among the members of the ensemble (from fully balanced (=1) to unbalanced (=0); *x*-axis). This is operationalized by simulating performances such that the phase coupling matrix is linearly interpolated between the actual data *α_i,j_* (triangle in figure 3*e*), the democratic matrix (*α*^balanced^*_i,j_*; square figure 3*e*) and no coupling (with zero coupling matrix; circle in figure 3*e*). This results in the following coupling matrix:

4.2 $$\alpha^{\mathrm{sim}}{\;}_{i,j}=y*((1-x)*\alpha_{i,j}\;+\;x*\alpha^{\mathrm{balanced}}{\;}_{i,j})\;+\;(1-y)*\alpha^{\mathrm{no}}{\;}_{i,j}.$$

## Data accessibility

Recording, raw data and processed data are available in an OSF repository: https://doi.org/10.17605/OSF.IO/8WYAV.
